# Acute Administration of URB597 Fatty Acid Amide Hydrolase Inhibitor Prevents Attentional Impairments by Distractors in Adolescent Mice

**DOI:** 10.3389/fphar.2019.00787

**Published:** 2019-07-19

**Authors:** Gabriella Contarini, Valentina Ferretti, Francesco Papaleo

**Affiliations:** Department of Neuroscience and Brain Technologies, Genetics of Cognition Laboratory, Istituto Italiano di Tecnologia, Genova, Italy

**Keywords:** 5-CSRTT, adolescence, fatty acid amide hydrolase, URB597, endocannabinoid system, anandamide, attentional control, cognitive functions

## Abstract

The maturation of attentional control during adolescence might influence later functional outcome or predisposition to psychiatric disorders. During adolescence, the cannabinoid system is particularly sensitive to pharmacological challenges, with potential impact on cognitive functions. Here, we used a recently validated five-choice serial reaction time task protocol to test adolescent C57BL/6J mice. We showed that the pharmacological inhibition (by URB597) of the fatty acid amide hydrolase (FAAH), the major enzyme implicated in anandamide degradation, prevented cognitive disruptions induced by distracting cues in adolescent mice. In particular, these protective effects were indicated by increased accuracy and correct responses and decreased premature responses selectively in the distractor trials. Notably, at the relatively low dose used, we detected no effects in other cognitive, motor, or incentive measures nor long-lasting or rebound effects of FAAH inhibition in cognitive functions. Overall, these data provide initial evidence of selective procognitive effects of FAAH inhibition in measures of attentional control in adolescent mice.

## Introduction

Adolescence is a critical period for the brain development, with the transition from childhood to adulthood influencing several aspects of mammalian behavior and especially cognitive functions (Spear, 2000; Schneider, 2013). One of the most critical functions influenced by the maturation of cortical area during adolescence is attentional control. Adolescents, in fact, usually show increased distractibility compared to adults when high levels of attention are required (Dumontheil et al., 2010). Notably, possibly linked with the drastic rearrangement of several neuronal systems (Spear, 2000; Galve-Roperh et al., 2009), alterations in executive functions, such as attentional control and cognitive liability to distractions during adolescence, have been associated with higher predisposition to psychiatric disorders (Spear, 2000). Thus, it is important to investigate the mechanisms that influence attentional control abilities during adolescence.

The cannabinoid system has been implicated in a number of different cognitive functions, including learning and memory processes (Qin et al., 2015; Lee et al., 2016; Scheggia et al., 2018). In particular, the cannabinoid system is more susceptible to pharmacological challenges during adolescence, with potential long-lasting effects in neuronal development and circuit connectivity in species including rodents and humans (Aguado et al., 2006; Berghuis et al., 2007; Harkany et al., 2007; Galve-Roperh et al., 2009; Cass et al., 2014; Maccarrone et al., 2016). In agreement, increasing evidence points to a possible deleterious impact of cannabis consumption during adolescence on cognitive functions that could have long-lasting effects later in life (Gruber et al., 2012; Morin et al., 2019) and could depend on the age of the onset of cannabis use (Pattij et al., 2008; Gruber et al., 2012; Cuttler and Spradlin, 2017). Similarly, preclinical studies demonstrated that chronic treatment with ∆^9^-tethrahydrocannabinol (∆^9^-THC), the main psychoactive compound of cannabis, has a more negative impact on cognitive functions such as working memory, object recognition, and prepulse inhibition in adolescent rodents compared to adults (Schneider and Koch, 2007; Quinn et al., 2008; Curran et al., 2016).

∆^9^-THC acting through the cannabinoid receptor 1 (CB1R) might modulate several cognitive functions (Quinn et al., 2008; Qin et al., 2015; Scheggia et al., 2018), including attention (Solowij et al., 2002). The endogenous ligands of CB1R are anandamide (AEA; Devane et al., 1992) and 2-arachidonoylglycerol (2-AG; Hanus et al., 2002), which have been implicated in functions such as neuroinflammation, anxiety, and depression (Jayamanne et al., 2006; Busquets-Garcia et al., 2011; Scalvini et al., 2016; Danandeh et al., 2018). Moreover, both AEA and 2-AG have been implicated in the regulation of learning and memory processes (Varvel et al., 2007; Mazzola et al., 2009; Zanettini et al., 2011; Llorente-Berzal et al., 2015; Morena et al., 2015; Hartley et al., 2016; Ratano et al., 2018). In particular, endogenous AEA is produced “on demand” and acts with retrograde mechanisms binding CB1R as a partial agonist (Hillard, 2000; Piomelli, 2003). Then, AEA is quickly degraded by the fatty acid amide hydrolase (FAAH; Piomelli et al., 2006). The exogenous administration of AEA has been shown to produce cognitive impairments in mice (Costanzi et al., 2004). In contrast, when AEA levels are increased via the pharmacological inhibition of the FAAH or by genetic FAAH deletion, cognitive abilities are improved (Piomelli et al., 2006; Varvel et al., 2007; Panlilio et al., 2013) even if studies reporting cognitive impairments after FAAH inhibition are also evident (Busquets-Garcia et al., 2011; Goonawardena et al., 2011). Moreover, similar to ∆^9^-THC, AEA administration *per se* can induce other adverse effects such as hypothermia, catalepsy, antinociception, and hypomotility (Blankman and Cravatt, 2013). In contrast, FAAH inhibition showed no such side effects (Kathuria et al., 2003; Piomelli et al., 2006). This evidence thus increased the interest in pharmacologically targeting the FAAH as a means to improve cognitive dysfunctions (Chicca et al., 2016). However, an initial clinical trial that involved healthy volunteers using the FAAH inhibitor BIA-10-2474 drastically failed due to the neurological side effects (Mallet et al., 2016; Moore, 2016). Successively, these side effects were ascribed to the high doses of the compound used and the consequent unselective effects of the FAAH inhibitor (Mallet et al., 2016; Moore, 2016). Further preclinical studies are then still needed to assess the efficacy and safety of FAAH inhibitors, especially if the target might be a critical period of development such as adolescence.

To start investigating the possible cognitive effects of FAAH inhibition during adolescence, overcoming possible ethical implications and confounding factors linked with human studies (e.g., genetic heterogeneity, environment, pathological state, and pharmacological treatments), here we assessed the impact of a controlled vehicle or URB597 exposure during adolescence in C57BL/6J in a modified five-choice serial reaction time task (5-CSRTT) for adolescent mice (Ciampoli et al., 2017). We selected URB597 because this is one of the most well-characterized FAAH inhibitors among the several compounds synthesized and tested (Kathuria et al., 2003; Fegley et al., 2005; Piomelli et al., 2006). We choose the 5-CSRTT task originally developed to mimic the human continuous performance task tests of Rosvold and Mirsky (Robbins, 2002), because it allows to simultaneously measure different parameters related to impulsivity, compulsivity, inattentiveness, speed of processing, motivational status, and cognitive vulnerability to distractors also in adolescent mice (Robbins, 2002; Ciampoli et al., 2017; Huang et al., 2017). Investigating the role of FAAH inhibition might be relevant considering that adolescents’ reduced attentional control and increased distractibility are identified as possible risk factors for the development of psychiatric diseases (Frame and Oltmanns, 1982; Slobodin et al., 2018).

## Materials and Methods

### Mice

**Figure 1:**
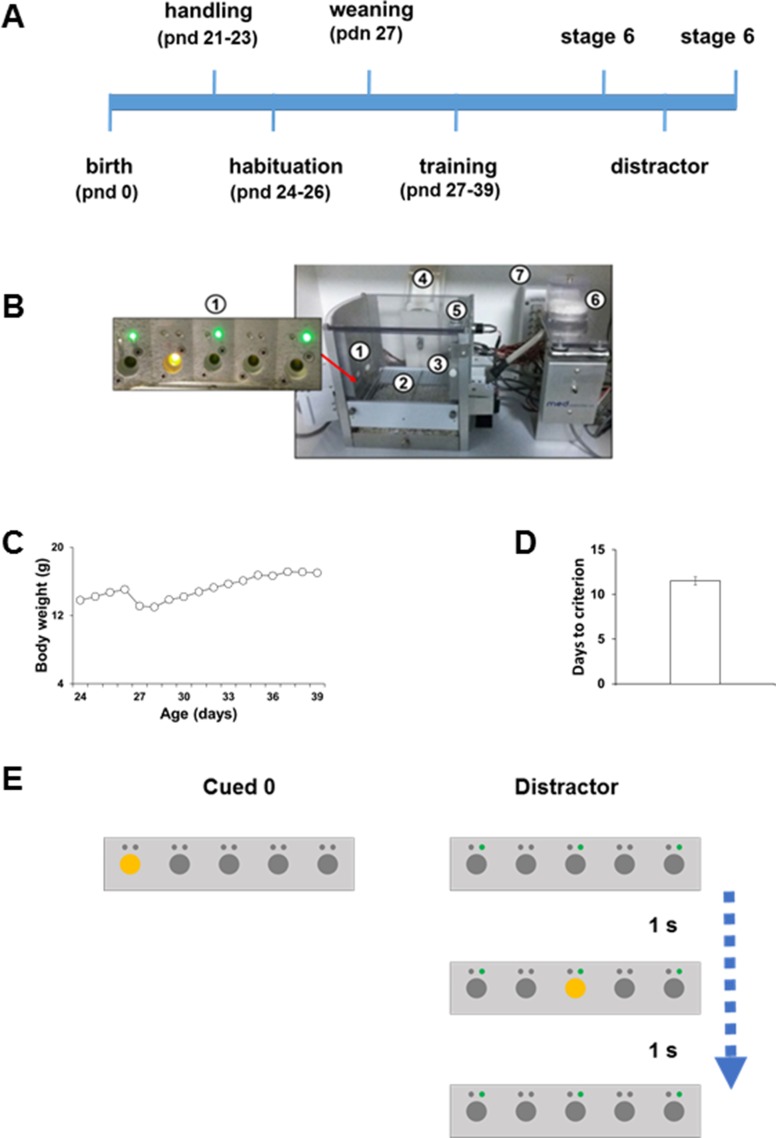
**(A)** Schematic timeline of experimental proctocol. **(B)** The modified 5CSRTT apparatus: (1) modified five nose-poke holes wall, each outfitted with a recessed LED stimulus light and an additional LED cue light (green) above each of the five nose-poke holes. (2) A stainless-steel grid floor modified for use in adolescent mice. (3) Food magazine on the wall opposite to the five-hole array. (4) Water dispenser. (5) House-light. (6) Food pellet dispenser. (7) Smart Control Panel. (All the standard components were obtained from Med Associates, St. Albans, VT, USA.) **(C)** Morning body weight measurements (in grams) of adolescent C57BL/6J. **(D)** Number of days taken by C57BL/6J adolescent mice to reach Stage 6 criteria. **(E)** Schematic diagrams of the types of trials that were presented to the mice during the modified distractor manipulation.

All procedures were approved by the Italian Ministry of Health (permit nos. 230/2009-B and 107/2015-PR) and the local animal use committee and were conducted in accordance with the Guide for the Care and Use of Laboratory Animals of the National Institutes of Health and the European Community Council Directives. Rodent adolescence is usually considered between postnatal days (PND) 28 and 45 (Adriani and Laviola, 2003; Schneider, 2013). We used in-house bred mice within the range of 21- to 45-day-old C57BL/6J (n=22). We tested male and female mice but did not find any sex differences between two groups nor during the training phase or testing phase (data not shown). Mice were weaned on PND 26 (**Figure 1A**) and housed two to four per cage in individually ventilated cages (Tecniplast). Mice were housed in a climate-controlled specific pathogen-free animal facility (22±2°C) and maintained on a 12 h light/dark cycle (lights on from 7 am to 7 pm). All behavioral tests were conducted during the dark phase of the cycle.

### Apparatus

Training and testing were conducted in 12 operant chambers (Med Associates, St. Albans, VT, USA) as described previously (**Figure 1B**; Ciampoli et al., 2017; Huang et al., 2017). Briefly, two strings of LED lights were installed onto the ceiling of each of the sound-attenuating boxes and controlled by a timer to ensure 12 h light/dark. Each operant chamber contained a five nose-poke hole wall outfitted with an LED stimulus light for each hole. Additional LED pre-cue lights were installed above each of the five nose-poke holes. An infrared beam detected nose-poke. On the opposite wall of the five holes, there was a food magazine and a head entry detector for food reinforcement. A water dispenser on the latter wall allowed the mice to have full access to water throughout the test. A houselight was located above the food magazine. The operant chambers were connected to a smart control panel and interfaced to a Windows computer equipped with an MED-PC V software (Med Associates).

### Habituation

Mice were daily exposed to 1 min handling sessions from PND 21 to 23 (**Figure 1A**). During the handling sessions, mice were weighed to obtain a baseline of their ad libitum body weight (**Figure 1C**). To habituate animals to the reward pellet flavor, ten 14 mg pellets of the 5TUL diet were added for each mouse in the home cage. To maintain at least 90% of the weight, “daytime food restriction” was imposed during the experiment, whereas water was available ad libitum. Mice were not given access to the food when in their holding cages unless they are losing weight. In this case, extra food was provided during the day to keep the mice at their normal body weight curve of adolescent growth. From PND 24 to 26, mice were daily habituated to the 5-CSRTT apparatus for 1 h/day (**Figure 1A**). After the habituation phase, mice were weaned on PND 26 (**Figure 1A**). Training was started on PND 27 (**Figure 1A**). When in the testing cage, mice received food in the form of pellets (5TUL Purified rodent tablet, Test Diet).

### Training Protocol

Training was performed as described previously (Ciampoli et al., 2017). Mice were daily placed into the operant chambers at 5 pm and taken out the following morning at 9 am. During the day, animals were housed in their regular home-cage. Each night, mice were exposed to three testing sessions presented with a variable delay between sessions (2–5 h). Mice were daily weighed (**Figure 1C**) in the morning immediately after being taken out of the apparatus. When a head entry was detected, the first trial began with an intertrial interval (ITI). Any nose-poke during the ITI was recorded as a premature response resulting in a time-out (TO) period with the houselight turned on. At the end of the TO period, the houselight was turned back off and the ITI was restarted. Any nose-poke during the TO period reset the TO period. At the end of the ITI, the program randomly selected a stimulus location (one of five stimulus lights) and turned on the corresponding stimulus light. The stimulus light remained on for the stimulus duration (SD) value set. The animal had limited hold (LH) time to nose-poke into the lit hole. A nose-poke into the lit hole during the LH time was recorded as a correct response, the stimulus light was turned off if not turned off earlier, and a food pellet was delivered in the opposite-wall food magazine. A nose-poke into any of the other apertures was recorded as an incorrect response. Errors resulted in the initiation of a 5 s TO phase, during which the houselight was switched on and all holes were unresponsive. A lack of response within the LH period was deemed as omission and resulted in a TO period and no reward. Premature responses (occurring in the ITI before presentation of the trigger light stimulus) also led to a TO without reward and to a resetting of the trial. A perseverative response was scored when mice continued to poke in the same response hole when it no longer stood for a correct choice. The time from the onset of the light stimulus to the performance of a correct nose-poke response and the time from the correct response to the retrieval of the food reward from the magazine were recorded as the correct latency and reward latency, respectively. Training consisted of six stages. To proceed to each subsequent stage, mice were required to reach the criterion for two consecutive sessions. Each stage was more challenging than the last, with the SD and LH period decreasing and the other criteria become more demanding (see below). Sessions ended after 30 min or 100 trials, whichever came first. The criteria to reach each subsequent stage are as follows:

1. Stage 1 to 2: SD=20 s; LH=30 s; ITI=2 s. Criteria: ≥20 correct trials; ≥20% correct.
2. Stage 2 to 3: SD=10 s; LH=30 s; ITI=2 s. Criteria: ≥30 correct trials; ≥30% correct.
3. Stage 3 to 4: SD=8 s; LH=20 s; ITI=5 s. Criteria: ≥40 correct trials; ≥80% accuracy; ≤60% omission.
4. Stage 4 to 5: SD=4 s; LH=10 s; ITI=5 s. Criteria: ≥40 correct trials; ≥80% accuracy; ≤60% omission.
5. Stage 5 to 6: SD=2 s; LH=7 s; ITI=5 s. Criteria: ≥45 correct trials; ≥80% accuracy; ≤60% omission.
6. Stage 6: SD=1 s; LH=7 s; ITI=5 s.

Upon reaching Stage 6 (**Figure 1D**), mice were subjected to an extra day of testing at Stage 6. After that, mice were tested in the distractor test (**Figure 1E**). To assess possible rebound or long-lasting effects of the drug, mice were retest in Stage 6 the day after URB597 administration (**Figure 1A**). The following parameters were recorded to assess task performance:

- Total responses: the number of total responses (correct, incorrect, premature, perseverative, and TO of responses)
- Percentage of accuracy: the number of correct responses divided by the sum of the number of correct and incorrect responses multiplied by 100.
- Percentage of premature responses: the number of premature responses divided by the sum of correct, incorrect, premature, perseverative, and TO responses (total number of responses) multiplied by 100.
- Percentage of omissions: the number of omissions divided by the total number of trials multiplied by 100.
- Percentage of correct responses: the number of correct responses divided by the total number of trials run multiplied by 100.
- Correct latency: the total time from the onset of light stimulus to the performance of a correct response divided by the number of correct responses.
- Percentage of incorrect responses: the number of incorrect responses divided by the total number of trials run multiplied by 100.
- Incorrect latency: the total time from the onset of light stimulus to the performance of an incorrect response divided by the number of incorrect responses.
- Percentage of perseverative responses: the number of perseverative responses divided by the total number of responses run multiplied by 100.
- Reward latency: the total time from the performance of a correct response to the retrieval of the food reward from the food magazine divided by the number of correct responses.

### Distractor Test

In this study, we used the implemented Distractor test version 2 as validated by Ciampoli et al. (2017) (**Figure 1E** for a representative scheme). In this manipulation, the Cued 0 trial (presented 80% of the time within a session) was the standard trial type as in Stage 6. The distractor trial randomly occurred 20% of the time and was the same as the Cued 0 trial with the addition of a flashing green pre-cue light over the nose-poke hole numbers 1, 3, and 5 turned on for 1 s before 1 s after the stimulus light duration. Any nose-poke that occurred before the normal stimulus light was presented was considered a premature response and was not rewarded, resulting in a TO period.

### Drug Treatment

URB597 (cyclohexyl carbamic acid 3′-carbamoyl-3-y1 ester) was purchased from Sigma-Aldrich and was prepared as described previously (Manduca et al., 2014). Briefly, URB597 was dissolved in a vehicle containing 5% Tween 80, 5% polyethylene glycol 400, and 90% saline. URB597 or vehicle solutions were administered intraperitoneally (i.p.) at a dose of 0.5 mg/kg, based on previous studies (Kathuria et al., 2003), 20 min before the first nocturnal session of the task.

### Statistical Analysis

Results are expressed as mean±standard error of the mean all throughout the study. Two-way analyses of variance (ANOVAs) with treatment as between-subjects factors and trial type as within-subject repeated measures were used to analyze each single parameter measured (Total response, %Correct, %Accuracy, %Omission, %TO, %Premature, %Perseverative, Correct latency, Incorrect latency, and Reward latency). Newman-Keul’s *post hoc* test with multiple comparisons corrections was used for making comparisons between groups when the overall ANOVA showed statistical significant differences for the main factors or interactions. The accepted value for significance was p<0.05. All statistical analyses were performed using Statistica version 12 software (Statistica, StatSoft, Inc.).

## Results

### Training of Adolescent Mice in the Modified 5-CSRTT Paradigm

Mice (27 days old) were manipulated and trained in the modified 5-CSRTT (**Figure 1B**) as described previously (**Figure 1A** for the experimental timeline; Ciampoli et al., 2017). In agreement with our previous evidence (Ciampoli et al., 2017), about 85% of the mice acquired the task with an average of 12 days (**Figure 1D**). Throughout the test, all mice kept on growing as normal during this developmental phase (**Figure 1C**).

**Figure 2:**
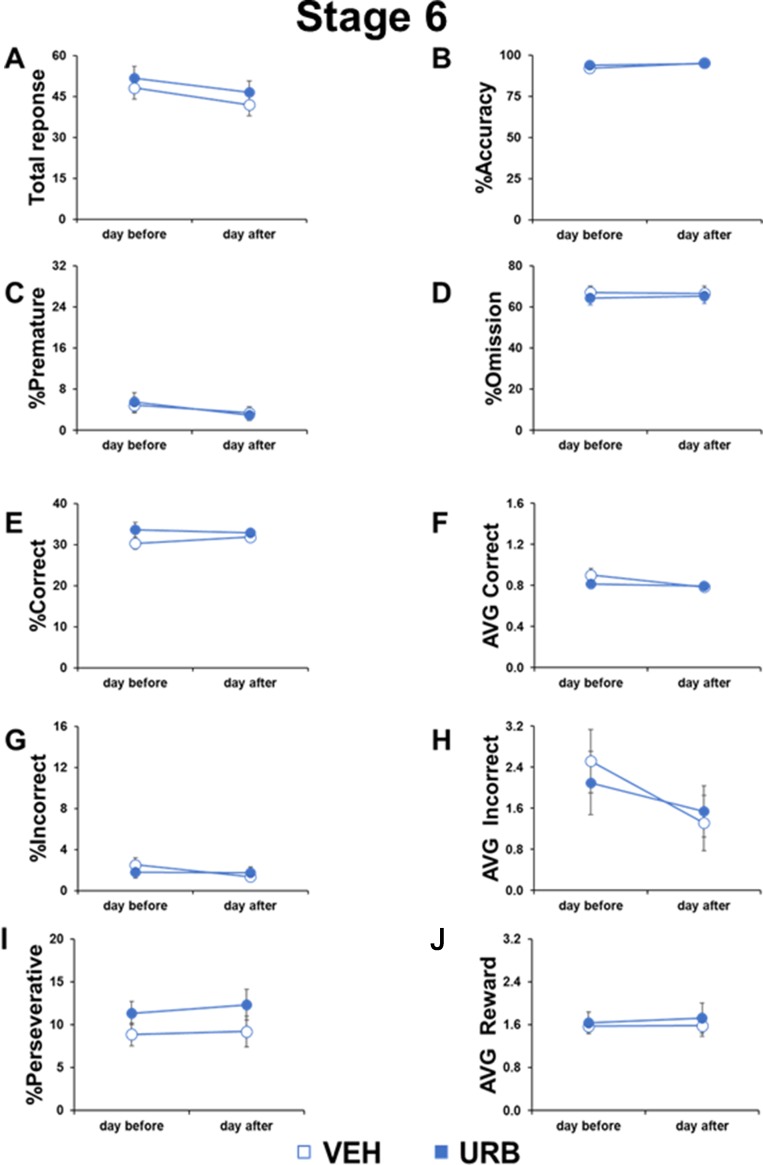
No permanent or residual effects of URB597 in adolescent mice cognitive responses. Different parameters measured during the 5-CSRTT in adolescent male mice treated with vehicle (n=11) or URB597 (n=11), 1 day before and 1 day after the Distractor test. **(A)** Number of total responses; **(B)** percentage of accuracy; **(C)** percentage of premature responses; **(D)** percentage of omitted responses; **(E)** percentage of corrct responses; **(F)** latency (in seconds) to correct response; **(G)** percentage of incorrect responses; **(H)** average to an incorrect response. **(I)** percentage of perseverative responses; **(J)** average to collect reward.

After reaching criteria, mice were then divided in two experimental groups with identical performance in each single parameter measured by the 5-CSRTT (see **Figure 2**: day before test).

### URB597 Prevented Detrimental Effects of Distractor Cues on Attentional Control in Adolescent Mice

**Figure 3:**
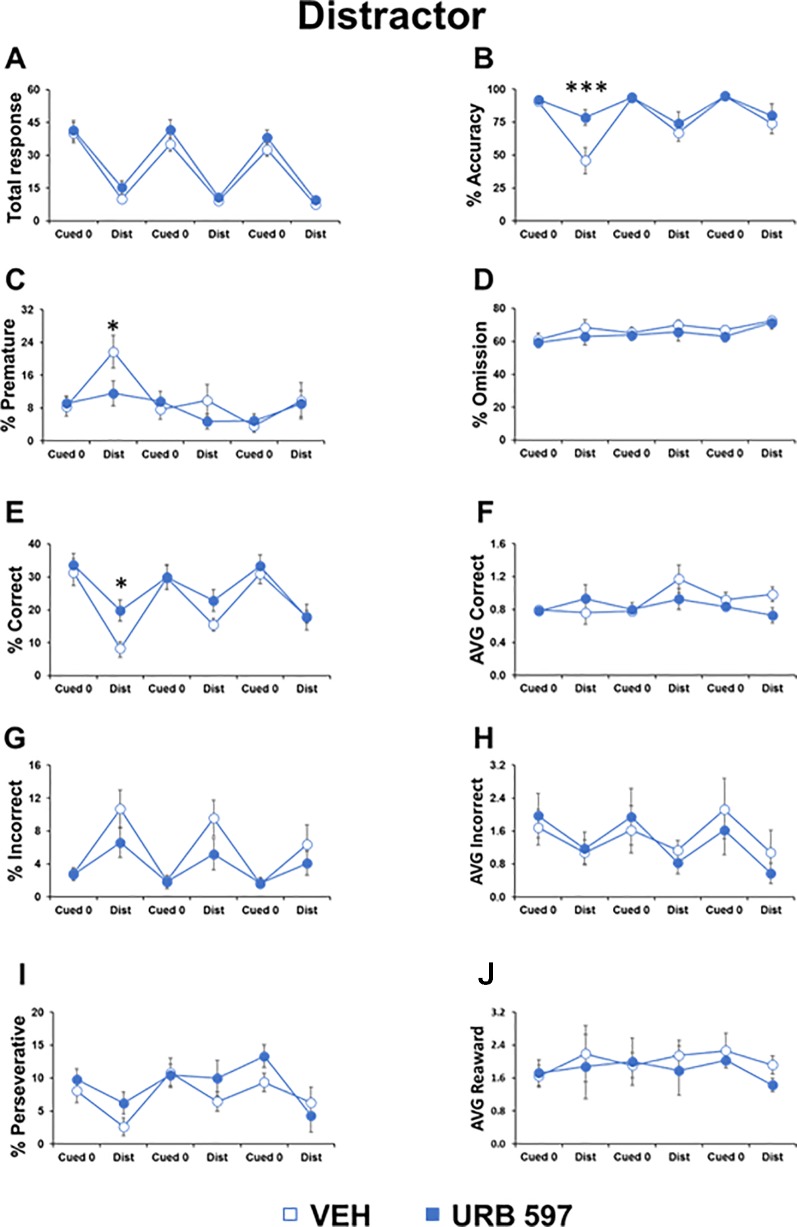
URB597 prevented the effects of distractor cues on attentional control in adolescent mice. Different parameters showed by adolescent male mice, treated with vehicle (n=11) or URB597 (n=11) during the Distractor test. **(A)** Number of total responses; **(B)** percentage of accuracy; **(C)** percentage of premature responses; **(D)** percentage of omitted responses; **(E)** percentage of correct responses; **(F)** latency (in seconds) to a correct response; **(G)** percentage of incorrect responses; **(H)** latency (in seconds) to an incorrect response; **(I)** percentage of perseverative responses; **(J)** latency (in seconds) to collect food from food magazine. *p<0.05, ***p<0.0005 versus vehicle.

To test the impact of URB597, we injected mice belonging to the two groups with URB597 or vehicle. We found that the URB597-treated group had higher accuracy (F_1,20_=9.842, p=0.0003) and increased correct responses (F_1,20_=4.620, p=0.03) compared to the vehicle-treated group, selectively in the distractor trials and only during the first night session performed 20 min after drug injection. In the same session, URB597 also prevented the increase of premature responses in the distractor trials (F_1,20_=3.803, p=0.0028; **Figure 3**). No differences were evident in all the other parameters and in the cognitive trials without distracting cues (“cued 0 trials”; **Figure 3**). Moreover, no effect of the URB597 challenge was evident in the second and third sessions performed 6 and 10 h after administration, respectively.

As found previously (Ciampoli et al., 2017), compared to the basic trial of the task, distracting cues decreased the total responses made (session 1: F_1,20_=104.901, p<0.0001; session 2: F_1,20_=148.686, p<0.0001; session 3: F_1,20_=06.612, p<0.0001) and decreased accuracy (session 1: F_1,20_=31.881, p=0.005; session 2: F_1,20_=5.169, p=0.0001; session 3: F_1,20_=9.039, p=0.007) and correct responses (session 1: F_1,20_=73.443, p<0.0001; session 2: F_1,20_=15.761, p=0.0007; session 3: F_1,20_=44.576, p<0.0001; **Figure 3**). In contrast, incorrect responses increased (session 1: F_1,20_=18.891, p=0.0003; session 2: F_1,20_=12.566, p=0.002; session 3: F_1,20_=7.068, p=0.383; **Figure 3**). Moreover, the distracting cues triggered more premature responses (session 1: F_1,20_=7.916, p=0.01) and less perseverative responses (session 1: F_1,20_=5.531, p=0.029; session 3: F_1,20_=4.713, p=0.0421; **Figure 3**) compared to their levels in trials without distracting cues.

Overall, these data demonstrated the ability of this paradigm to measure the deleterious impact of distracting cues in attentional control in adolescent mice and a specific effect of URB597 in preventing these cognitive disruptions.

### No Permanent or Residual Effects of URB597 in Cognitive Responses in Adolescent Mice

To assess possible residual or rebound effects of URB597 treatment, we compared the cognitive performance of adolescent mice in the 5-CSRTT the day before and the day after the URB597 challenge.

No day-by-treatment effect was evident in any parameter measured (**Figure 2**), including total responses (F_1,20_=0.0357, p=0.8521), accuracy (F_1,20_=1.060, p=0.3154), premature responses (F_1,20_=0.5989, p=0.4480), omitted responses (F_1,20_=0.8973, p=0.3548), correct responses (F_1,20_=1.7847, p=0.1966), correct latency (F_1,20_=4.176, p=0.0543), incorrect responses (F_1,20_=3.2227, p=0.0875), incorrect latency (F_1,20_=0.9804, p=0.3339), perseverative responses (F_1,20_=0.2260, p=0.6396), and reward latency (F_1,20_=0.0934, p=0.7631). However, compared to the day before the URB597 challenge, the day after the injection, mice made less total responses (F_1,20_=4.097, p=0.05), less premature responses (F_1,20_=9.0577, p=0.0069), and faster correct responses (F_1,20_=9.097, p=0.0068) and incorrect responses (F_1,20_=7.1016, p=0.0149) in an URB597-independent way. These data indicated that mice generally kept on improving their performance with repeated testing and that URB597 treatment did not have any long-lasting or residual effects in attentional control.

## Discussion

The main finding of this study is that acute exposure to 0.5 mg/kg URB597 in adolescence prevented the detrimental effects of distracting cues in attentional control while having no effects on general measures of cognitive and reward functioning.

The cognitive-protective effects of URB597 in adolescent mice were demonstrated by a selective increase in accuracy and correct responses and decrease in premature responses only in the distractor trials of our modified 5-CSRTT protocol. The role of the endocannabinoid system in the 5-CSRTT was previously investigated by Pattij et al. (2008) using both WIN 55,212-2, a CB1R synthetic agonist, and rimonabant, a synthetic antagonist of CB1R in adult rats. WIN 55,212-2 did not affect the 5-CSRTT performance, whereas rimonabant tended to improve attentional performance in the 5-CSRTT as shown by a slight increase in the percentage of correct responses and a decrease in the number of premature responses (Pattij et al., 2007). However, to our knowledge, there are no reports investigating the effects of FAAH inhibition in the 5-CSRTT or in adolescent rodents.

The effects of an acute administration of URB597 in attentional processes have been previously tested in the delay non-matched to the sample operant task, where decreased correct responses or no effects have been reported (Goonawardena et al., 2011; Panlilio et al., 2016). The reasons of the discrepancy with our data might be related to a number of different factors including the different tasks used or the different rodents species/strains (e.g., rats or mice). However, another important factor to consider might be the dose of the drug used (i.e., 3 mg/kg in previous studies versus 0.5 mg/kg used in the current study). In fact, it is not surprising that drugs acting on the cannabinoid system might have contrasting effects in cognitive functions depending on the dose. For instance, high doses of ∆^9^-THC have been shown to produce memory impairment, whereas low doses ameliorated memory deficits in a model of Alzheimer’s disease (Calabrese and Rubio-Casillas, 2018). Relative low doses of URB597 (0.1–1 mg/kg) have been reported to produce cognitive improvements in a passive avoidance task (Mazzola et al., 2009) but cognitive impairments in an object recognition task and a context recognition task (Busquets-Garcia et al., 2011). Our choice of the 0.5 mg/kg dose was based on previous studies (Kathuria et al., 2003; Mazzola et al., 2009) to avoid altered locomotor and stereotyped behaviors (“jump episodes”) induced by the lower 0.1 mg/kg dose but not evident from 0.3 to 1 mg/kg (Mazzola et al., 2009). However, we acknowledge that to better understand URB597 impact in attentional control a range of different doses should be compared. Thus, our study constitutes an initial step investigating the effects of FAAH inhibition in attentional control during adolescence, which will require further analyses.

An important distinction between our study and the previous ones relied on the age of treatment. As extensively reported, the endocannabinoid system undergoes dynamic changes throughout development (Schneider, 2008; Caballero et al., 2016; Meyer et al., 2018). In rodents, brain CB1R reaches the highest concentration at the onset of adolescence and then starts to decrease in later stages of life (Schneider, 2008; Caballero et al., 2016). Similarly, FAAH expression in mice showed a clear transient increase from PND 35 to 45, particularly in brain areas implicated in attentional control such as the prefrontal cortex (Gee et al., 2015). This evidence highlighted that FAAH inhibition during adolescence might have a different functional relevance compared to later stages. Our results are in line with the view that a challenge to the cannabinoid system must consider the developmental stage as a critical factor and does motivate additional analyses designed to directly compare URB597 effects when given during adolescence or adulthood. Indeed, our findings provide an initial exploration on the role of FAAH inhibition during adolescence.

The URB-dependent cognitive effects we observed in adolescent mice were evident only in the first session performed 20 min after injection but not in the following sessions neither in the day after. This might seem in contrast to the evidence that FAAH inhibition induced by 0.3 mg/kg URB597 can reach a maximum effect within 15 min and persist for at least 16 h (Piomelli et al., 2006). However, the URB597-dependent increase in AEA concentrations reached its peak between 30 min and 2 h from the administration and then started to drastically decrease (Fegley et al., 2005; Piomelli et al., 2006). This is in agreement with our behavioral data, as the second session with the distractor trials, where we did not see URB597 effects, was performed on average 5 h after its injection. However, it should be considered that the lack of URB597 effects in our second and third nightly sessions might be also related to the partially reduced detrimental effects of distracting cues in these sessions compared to the first session. Nevertheless, future studies should address the potential long-lasting effects of prolonged treatment with URB597 during adolescence. Indeed, a report indicated that subchronic treatment with URB597 in adolescent rats might result in decreased CB1R levels still evident in adulthood in several brain regions implicated in cognition, such as the striatum, ventral tegmental area, and the hippocampus (Marco et al., 2008).

In this report, we did not directly assess the mechanisms underlying the behavioral effects of the 0.5 mg/kg URB597 dose adopted. A number of previous studies indicated that the effects induced by URB597 treatment are mediated by an action through CB1Rs (Kathuria et al., 2003; Jayamanne et al., 2006; Mazzola et al., 2009; Busquets-Garcia et al., 2011; Heinz et al., 2017; Danandeh et al., 2018). This has been confirmed using different behavioral tests and doses, suggesting that the effects of FAAH inhibition resulting in the enhancement of AEA levels might be mediated by CB1Rs. However, we cannot exclude that the cognitive effects we revealed might also involve other receptors such as peroxisome proliferator-activated receptor or transient receptor potential vanilloid (TRPV1) as suggested by other studies (Mazzola et al., 2009; Gobira et al., 2017). This might be the topic for a dedicated study.

In humans, deficits in attentional control are the core symptoms of several psychiatric disorders, especially attention-deficit hyperactivity disorder (ADHD). Among adolescent patients affected by ADHD, cannabis is one of the drugs used as auto-medication, because it might improve sleep and might help to maintain focused attention (Gudjonsson et al., 2012). This brought to test the effects of cannabis in adults by a randomized controlled trial (Cooper et al., 2017). An improvement in the symptom domains of ADHD was noticed; however, adverse events such as muscular seizures/spasms, feeling of lightheadedness, and sleep difficulties were also reported. Thus, in light of our new data, it might be tempting to speculate that the FAAH inhibition *per se* as well as FAAH inhibition with concomitant blockade of AEA uptake and/or TRPV1 channels might be a more efficient approach to improve cognitive dysfunctions in ADHD (Tzavara et al., 2006), with reduced side effects compared to those of cannabis or ∆^9^-THC.

In conclusion, our findings provide new insights about the impact of FAAH inhibition, and correlated cannabinoid system modulation, during adolescence in attentional control. Notably, at least when used acutely and at the relatively low dose chosen, URB597 treatment showed a selective ability to prevent the detrimental cognitive effects of distractors showing no side effects that could influence general cognitive performance.

## Data Availability

The data supporting the present study are available from the corresponding author on reasonable request.

## Ethics Statement

All procedures were approved by the Italian Ministry of Health (permits n. 230/2009-B and 107/2015-PR) and local Animal Use Committee and were conducted in accordance with the Guide for the Care and Use of Laboratory Animals of the NIH and the European Community Council Directives.

## Author Contributions

GC and FP contributed to the conceptualization. GC, VF, and FP contributed to the methodology and investigation. FP provided the resource. GC, VF, and FP wrote the manuscript. GC and FP performed the visualization and analysis. Supervision was done by FP. All of the authors revised the manuscript.

## Funding

This work was supported by funding from the Istituto Italiano di Tecnologia, the Brain and Behavior Research Foundation (2015 NARSAD n. 23234), and the Compagnia di San Paolo (2015-0321).

## Conflict of Interest Statement

The authors declare that the research was conducted in the absence of any commercial or financial relationships that could be construed as a potential conflict of interest.
